# Supplementary material for: Heat adaptation of phage T7 under an extended genetic code
Source: Virus Evol. 2021 Dec 1;7(2):veab100. doi: 10.1093/ve/veab100 (PMC8923235; doi:10.1093/ve/veab100)
Supplement: veab100_Supp [file veab100_supp.zip › Sequencing_Analysis.zip!/Sequencing_Analysis/S2/evidence/JC_SIDE_1_71.html]

BRESEQ :: Evidence


| New junction evidence | | | | | | | | | | | |
| --- | --- | --- | --- | --- | --- | --- | --- | --- | --- | --- | --- |
|  | | seq id | position | reads (cov) | reads (cov) | score | skew | freq | annotation | gene | product |
| \* | ? | NC\_001604.1 | = 19585 | 4495 (0.850) | 324 (0.060) | 168/586 | 12.2 | 6.9% | intergenic (–/–) | *–/–* | –/– |
|  | ? | NC\_001604.1 | 19598 = | 4417 (0.850) |  |  |  |  | intergenic (–/–) | *–/–* | –/– |
| Rejected: Coverage evenness skew score above cutoff. | | | | | | | | | | | |
| Rejected: Frequency below/above cutoff threshold. | | | | | | | | | | | |

Only 100 of 4803 total aligned reads displayed.  

`GTACATTCTGGACTCTACCGGAAAACATGTAGCGTACTTCGCGTGGTGCGTAAGCTGTGACATTCACCACAAAGGAGACATTCTGGATGTAACGTCCGTTGTCATTAATCCTGAGGCAGACTCTAAGGGCTTACAGCGATTCCTAGCGAAACGCTTTAAGTACCTTGCGGAACTCCACGATTGCGATTGGGTGTCTCGTTGTAAGCATGAAGGCGAGACAATGCGTGTATACTTTAAGGAGGTATAAGTTATGGGTAAGAAAGTTAAGAAGGCCGTGAAGAAAGTCACCAAGTCCGTTAAGAAAGTCGTTAAGGAAGGGGCTCGTCCGGTTAAACAGGTTGCTGGCGGTCTAGCTGGTCTGGCTGGTGGTACTGGTGAAGCACAGATGGTGGAAGTACCACAAGCTGCCGCACAGATTGTTGACGTACCTGAGAAAGAGGTTTCCACTGAGGACGAAGCACAGACAGAAAGCGGACGCAAGAAAGCTCGTGCTGGCGGTAAGAAATCCTTGAGTGTAGCCCGTAGCTCCGGTGGCGGTATCAACATTTAATCAGGAGGTTATCGTGGAAGACTGCATTGAATGGACCGGAGGTGTCAACTCTAAGGG  >  NC_001604.1/19285‑19891`  

                                                                                                                                                                                                                                                                                                            `|`                                                                                                                                                                                                                                                                                                                    
            `ctctACCGGAAAACATGTAGCGTACTTCGCGTGGTGCGTAAGCTGTGACATTCACCACAAAGGAGACATTCTGGATGTAACGTCCGTTGTCATTAATCCTGAGGCAGACTCTAAGGGCTTACAGCGATTCCTAGCGAAACGCTTTAAGTACCTTGCGGAACTCCACGATTGCGATTGGGTGTCTCGTTGTAAGCATGAAGGCGAGACAATGCGTGTATACTTTAAGGAGGTATAAGTTATGGGTAAGAAAGTTAAGAAGGCCGTGAAGAAAGTCACCAAGTACGTTAAGAAAGTCGTTaag                                                                                                                                                                                                                                                                                                        <  1:231682/301‑1 (MQ=255)`  
                `aCCGGAAAACATGTAGCGTACTTCGCGTGGTGCGTAAGCTGTGACATTCACCACAAAGGAGACATTCTGGATGTAACGTCCGTTGTCATTAATCCTGAGGCAGACTCTAAGGGCTTACAGCGATTCCTAGCGAAACGCTTTAAGTACCTTGCGGAACTCCACGATTGCGATTGGGTGTCTCGTTGTAAGCATGAAGGCGAGACAATGCGTGTATACTTTAAGGAGGTATAAGTTATGGGTAAGAAAGTTAAGAAGGCCGTGAAGAAAGTCACCAAGTCCGTTAAGAAAGTCGTTaaggaag                                                                                                                                                                                                                                                                                                    >  1:405822/1‑301 (MQ=255)`  
                     `aaaaCATGTAGCGTACTTCGCGTGGTGCGTAAGCTGTGACATTCACCACAAAGGAGACATTCTGGATGTAACGTCCGTTGTCATTAATCCTGAGGCAGACTCTAAGGGCTTACAGCGATTCCTAGCGAAACGCTTTAAGTACCTTGCGGAACTCCACGATTGCGATTGGGTGTCTCGTTGTAAGCATGAAGGCGAGACAATGCGTGTATACTTTAAGGAGGTATAAGTTATGGGTAAGAAAGTTAAGAAGGCCGTGAAGAAAGTCACCAAGTCCGTTAAGAAAGTCGTTAAGGAAGGGGcg                                                                                                                                                                                                                                                                                               >  1:21625/1‑300 (MQ=255)`  
                       `aaCATGTAGCGTACTTCGCGTGGTGCGTAAGCTGTGACATTCACCACAAAGGAGACATTCTGGATGTAACGTCCGTTGTCATTAATCCTGAGGCAGACTCTAAGGGCTTACAGCGATTCCTAGCGAAACGCTTTAAGTACCTTGCGGAACTCCACGATTGCGATTGGGTGTCTCGTTGTAAGCATGAAGGCGAGACAATGCGTGTATACTTTAAGGAGGTATAAGTTATGGGTAAGAAAGTTAAGAAGGCCGTGAAGAAAGTCACCAAGTCCGTTAAGAAAGTCGTTAAGGAAGGGGCTCg                                                                                                                                                                                                                                                                                             >  1:148862/1‑301 (MQ=255)`  
                            `gTAGCGTACTTCGCGTGGTGCGTAAGCTGTGACATTCACCACAAAGGAGACATTCTGGATGTAACGTCCGTTGTCATTAATCCTGAGGCAGACTCTAAGGGCTTACAGCGATTCCTAGCGAAACGCTTTAAGTACCTTGCGGAACTCCACGATTGCGATTGGGTGTCTCGTTGTAAGCATGAAGGCGAGACAATGCGTGTATACTTTAAGGAGGTATAAGTTATGGGTAAGAAAGTTAAGAAGGCCGTGAAGAAAGTCACCAAGTCCGTTAAGAAAGTCGTTAAGGAAGGGGCTCGTCCgg                                                                                                                                                                                                                                                                                        >  1:38350/1‑301 (MQ=255)`  
                              `aGCGTACTTCGCGTGGTGCGTAAGCTGTGACATTCACCACAAAGGAGACATTCTGGATGTAACGTCCGTTGTCATTAATCCTGAGGCAGACTCTAAGGGCTTACAGCGATTCCTAGCGAAACGCTTTAAGTACCTTGCGGAACTCCACGATTGCGATTGGGTGTCTCGTTGTAAGCATGAAGGCGAGACAATGCGTGTATACTTTAAGGAGGTATAAGTTATGGGTAAGAAAGTTAAGAAGGCCGTGAAGAAAGTCACCAAGTCCGTTAAGGAAggggctcgtccggttaaacaggttgcc                                                                                                                                                                                                                                                                                      >  1:467949/1‑275 (MQ=255)`  
                                  `tACTTCGCGTGGTGCGTAAGCTGTGACATTCACCACAAAGGAGACATTCTGGATGTAACGTCCGTTGTCATTAATCCTGAGGCAGACTCTAAGGGCTTACAGCGATTCCTAGCGAAACGCTTTAAGTACCTTGCGGAACTCCACGATTGCGATTGGGTGTCTCGTTGTAAGCATGAAGGGGAGACAATGCGTGTATACTTTAAGGAGGTATAAGTTATGGGTAAGAAAGTTAAGAAGGCCGTGAAGAAAGTCACCAAGTCCGTTAAGAAAGTCGTTAAGGAAGGGGCTCGTCCGGTTAAAc                                                                                                                                                                                                                                                                                  <  1:824351/301‑1 (MQ=255)`  
                                    `cTTCGCGTGGTGCGTAATCTGTGACATTCACCCCAAAGGGGACATTCTGGATGTAACGTCCGTTGTCATTAATCCTGAGGCAGACTCTAAGGGCTTACAGCGATTCCTAGCGAAACGCTTTAAGTACCTTGCGGAACTCCACGATTGCGATTGGGTGTCTCGTTGTAAGCATGAAGGCGAGACAATGCGTGTATACTTTAAGGAGGTATAAGTTATGGGTAAGAAAGTTAAGAAGGCCGTGAAGAAAGTCACCAAGTCCGTTAAGAAAGTCGTTAAGGAAGGGGCTCGTCCGGTTAAACAg                                                                                                                                                                                                                                                                                <  1:199880/301‑1 (MQ=255)`  
                                    `cTTCGCGTGGTGCGTAAGCTGTGACATTCACCACAAAGGAGACATTCTGGATGTAACGTCCGTTGTCATTAATCCTGAGGCAGACTCTAAGGGCTTACAGCGATTCCTAGCGAAACGCTTTGAGTACCTTGCGGAACTCCACGATTGCGATTGGGTGTCTCGTTGTAAGCATGAAGGCGAGACAATGCGTGTATACTTTAAGGAGGTATAAGTTATGGGTAAGAAAGTTAAGAAGGCCGTGAAGAAAGTCACCAAGTCCGTTAAGAAAGTCGTTAAGGAAGGGGCTCGTCCGGTTAAACAg                                                                                                                                                                                                                                                                                >  1:360341/1‑301 (MQ=255)`  
                                    `attCGCGTGGTGCTAAAGCTGTGACATTCACCACAAAGGAGACATTCTGGATGTAACGTCCGTTGTCATTAATCCTGAGGCAGACTCTAAGGGCTTACAGCGATTCCTAGCGAAACGCTTTAAGTACCTTGCGGAACTCCACGATTGCGATTGGGTGTCTCGTTGTAAGCATGAAGGCGAGACAATGCGTGTATACTTTAAGGAGGTATAAGTTATGGGTAAGAAAGTTAAGAAGGCCGTGAAGAAAGTCACCAAGTCCGTTAAGAAAGTCGTTAAGGAAGGGGCTCGTCCGGTTAAACAg                                                                                                                                                                                                                                                                                <  1:186513/300‑1 (MQ=255)`  
                                              `tgCGTAAGCTGTGACATTCACCACAAAGGAGACATTCTGGATGTAACGTCCGTTGTCATTAATCCTGAGGCAGACTCTAAGGGCTTACAGCGATTCCTAGCGAAACGCTTTAAGTACCTTGCGGAACTCCACGATTGCGATTGGGTGTCTCGTTGTAAGCATGAAGGCGAGACAATGCGTGTATACTTTAAGGAGGTATAAGTTATGGGTAAGAAAGTTAAGAAGGCCGTGAAGAAAGTAACCAAGTCGGTTAGGAAAGTCGTTAGGGAAGGGGCTCGTCCGGTTAAACGGGTTGCTGGCg                                                                                                                                                                                                                                                                      >  1:736950/1‑301 (MQ=255)`  
                                               `gCGTAAGCTGTGACATTCACCACAAAGGAGACATTCTGGATGTAACGTCCGTTGTCATTAATCCTGAGGCAGACTCTAAGGGCTTACAGCGATTCCTAGCGAAACGCTTTAAGTACCTTGCGGAACTCCACGATTGCGATTGGGTGTCTCGTTGTAAGCATGAAGGCGAGACAATGCGTGTATACTTTAAGGAGGTATAAGTTATGGGTAAGAAAGTTAAGAAGGCCGTGAAGAAAGTCACCAAGTCCGTTAAGAAAGTCGTTAAGGAAGGGGCTctgtctcttatacacatctccgagcc                                                                                                                                                                                                                                                                     >  1:779802/1‑276 (MQ=255)`  
                                               `gCGTAAGCTGTGACATTCACCACAAAGGAGACATTCTGGATGTAACGTCCGTTGTCATTAATCCTGAGGCAAACTCTAAGGGCTTACAGCGATTCCTAGCGAAACGCTTTAAGTACCTTGCGGAACTCCACGATTGCGATTGGGTGTCTCGTTGTAAGCATGAAGGCGAGACAATGCGTGTATACTTTAAGGAGGTATAAGTTATGGGTAAGAAAGTTAAGAAGGCCGTGAAGAAAGTCACCAAGTCCGTTAAGAAAGTCGTTAAGGAAGGGGCTCGTCCGGTTAAACAGGTTGCTGGCgg                                                                                                                                                                                                                                                                     >  1:321042/1‑301 (MQ=255)`  
                                                    `aGCTGTGACATTCACCACAAAGGAGACATTCTGGATGTAACGTCCGTTGTCATTAATCCTGAGGCAGACTCTAAGGGCTTACAGCGATTCCTAGCGAAACGCTTTAAGTACCTTGCGGAACTCCACGATTGCGATTGGGTGTCTCGTTGTAAGCATGAAGGCGAGACAATGCGTGTATACTTTAAGGAGGTATAAGTTATGGGTAAGAAAGTTAAGAAGGCCGTGAAGAAAGTCACCAAGTCCGTTAAGAAAGTCGTTAAGGAAGGGGCTCGTCCGGTTAAACAGGTTGCTGGCGGTCTAg                                                                                                                                                                                                                                                                >  1:84345/1‑301 (MQ=255)`  
                                                      `cTGTGACATTCACCACAAAGGAGACATTCTGGATGTAACGTCCGTTTTCATTAATCCTGAGGCAGACTCTAAGGGCTTACAGCGATTCCTAGCGAAACGCTTTAAGTACCTTGCGGAACTCCACGATTGCGATTGGGTGTCTCGTTTTAAGCATGAAGGCGAGACAATGCGTGTATACTTTAAGGAGGTATAAGTTATGGGTAAGAAAGTTAAGAAGGCCGTGAAGAAAGTCACCAAGTCCGTTAAGAAAGTCGTTAAGGAAGGGGCTCGTCCGGTTAAACAGGTTGCTGGCGGTCTAGCt                                                                                                                                                                                                                                                              <  1:608645/301‑1 (MQ=255)`  
                                                      `cTGTGACATTCACCACAAAGGAGACATTCTGGATGTAACGTCCGTTGTTATTACTCCTGAGGCAGACTCTAAGGGCTTTCAGCGATTCCTAGAGAAACGTTTTAAGTACCTTGCGGAACCCCTCGCTTGCGATTGGGTTTCTCCTTGTAAGCATGAAGCCGAGACAATCCGTCTATACTTTACGGCGGTATAAGTAATTGGTTAGAATGTTAAGAAGGCCGTGAAGAACGTCACCAAGTCCGTCAATCAAGTCGTTAACGACGGGGCCCGCCCTGTTAAACAGTTTGCTGGCCGTCTAGcc                                                                                                                                                                                                                                                              >  1:223431/1‑300 (MQ=255)`  
                                                          `gACATTCACCACAAAGGAGACATTCTTGATGTAACGTCCGTTGTCATTAATCCGGAGGCAGACTCTAAGGGCTTACAGCGATTCCTAGCGAAACGCTTTAAGTACCTTGCGGAACTCCACAATTGCGATTGGGTGTCTCGTTGTAAGCATGAAGGCGAGACAAGGCGTGTATATTTTAAGGAGGTATAAGTTATGGGTAAGAAAGTTAAGAAGGCCGTGAAGAAAGTCACCAAGTCCGTTAAGAAAGTCGTTAAGGAAGGGGCTCGTCCGGTTAAACAGGTTGCTGGCGGTCTAGCTGGTc                                                                                                                                                                                                                                                          <  1:341486/301‑1 (MQ=255)`  
                                                          `aaCATTCACCCCAAAGGAAACATTCTGGATGTAACGTCCGTTGTCATTATTCCTGAGGCAGACTCTAAGGGCTTACAGCGATTCCTAGCGAAACGCTTTAAGTACCTTGCGGAACTCCACGATTGCGATTGGGTGTCTCGTTGTAAGCATGAAGGCGAGACAATGCGTGTATACTTTAAGGAGGTATAAGTTATGGGTAAGAAAGTTAAGAAGGCCGTGAAGAAAGTCACCAAGTCCGTTAAGAAAGTCGTTAAGGAAGGGGCTCGTCCGGTTAAACAGGTTGCTGGCGGTCTAGCTGGTc                                                                                                                                                                                                                                                          <  1:258206/300‑1 (MQ=255)`  
                                                             `tataagagacagggAGACATTCTGGATGTAACGTCCGTTGTCATTAATCCTGAGGCAGACTCTAAGGGCTTACAGCGATTCCTAGCGAAACGCTTTAAGTACCTGGCGGAACTCCACGATTGCGATTGGGTGTCTCGTTGTAAGCATGAAGGCGAGACAATGCGGGTATACTTTAAGGAGGTATAAGTTATGGGTAAGAAAGTTAAGAAGGCCGTGAAGAAAGTCACCAAGTCCGTTAAGAAAGTCGTTAAGGAAGGGGCTCGTCCGGTTAAACAGGTTGCTGGCGGTCTAGCTGGtggta                                                                                                                                                                                                                                                       <  1:593864/289‑5 (MQ=255)`  
                                                             `aTTCACCACAAAGGAGACATTCTGTATGTAACGTCCGTTGTCATTAATCCTGAGGCAGACTCTAAGGGCTTACAGCGATTCCTAGCGAAACGCTTTAAGTACCTTGCGGAACTCCACGATTGCGATTGGGTGTCTCGTTGTAAGCATGAAGGCGAGACAATGCGTGTATACTTTAAGGAGGTATAAGTTATGGGTAAGAAAGTTATGAAGGCCGTGAAGAAAGTCACCAAGTCCGTTAAGAAAGTCGTTAAGGAAGGGGCTCGTCCGGTTAAACAGGTTGCTGGCGGTCTAGCTGGTctgg                                                                                                                                                                                                                                                       >  1:29097/1‑301 (MQ=255)`  
                                                               `tCACCACAAAGGAGACATTCTGGATGTAACGTCCGTTGTCATTAATCCTGAGGCAGACTCTAAGGGCTTACAGCGATTCCTAGCGAAACGCTTTAAGTACCTTGCGGAACTCCACGATTGCGATTGGGTGTCTCGTTGTAAGCATGAAGGCGAGACAATGCGTGTATACTTTAAGGAGGTATAAGTTATGGGTAAGAAAGTTAAGAAGGCCGTGAAGAAAGTCACCAAGTCCGTTAAGAAAGTCGTTAAGGAAGGGGCTCGTCCGGTTAAACAGGTTGCTGGCGGTcctgtctcttataca                                                                                                                                                                                                                                                     >  1:460726/1‑287 (MQ=255)`  
                                                                `ggagatgtgtataagagacaggATGTAACGTCCGTTGTCATTAATCCTGAGGCAGACTCTAAGGGCTTACAGCGATTCCTAGCGAAACGCTTTAAGTACCTTGCGGAACTCCACGATTGCGATTGGGTGTCTCGTTGTAAGCATGAAGGCGAGACAATGCGTGTATACTTTAAGGAGGTATAAGTTATGGGTAAGAAAGTTAAGAAGGCCGTGAAGAAAGTCACCAAGTCCGTTAAGAAAGTCGTTAAGGAAGGGGCTCGTCCGGTTAAACAGGTTGCTGGCGGTCTAGCTGGTctggctg                                                                                                                                                                                                                                                    <  1:772178/281‑1 (MQ=255)`  
                                                                  `ccacAAAGGAGACATTCTGGATGTAACGTCCGTTGTCATTAATCCTGAGGCAGACTCTAAGGGCTTACAGCGATTCCTAGCGAAACGCTTTAAGTACCTTGCGGAACTCCACGATTGCGATTGGGTGTCTCGTTGTAAGCATGAAGGCGAGACAATGCGTGTATACTTTAAGGAGGTATAAGTTATGGGTAAGAAAGTTAAGAAGGCCGTGAAGAAAGTCACCAAGTCCGTTAAGAAAGTCGTTAAGGAAGGGGCTCGTCCGGTTAAACAGGTTGCTGGCGGTCTGGCTGGtggtactggt                                                                                                                                                                                                                                                  >  1:74402/1‑292 (MQ=255)`  
                                                                  `ccacAAAGGAGACATTCTGGATGTAACGTCCGTTGTCATTAATCCTGAGGCAGACTCTAAGGGCTTACAGCGATTCCTAGCGAAACGCTTTAAGTACCTTGCGGAACTCCACGATTGCGATTGGGTGTCTCGTTGTAAGCATGAAGGCGAGACAATGCGTGTATACTTTAAGGAGGTATAAGTTATGGGTAAGAAAGTTAAGAAGGCCGTGAAGAAAGTCACCAAGTCCGTTAAGAAAGTCGTTAAGGAAGGGGCTCGTCCGGTTAAACAGGTTGCTGGCGGTCTAGCTGGTCTGGCtggt                                                                                                                                                                                                                                                  >  1:648098/1‑301 (MQ=255)`  
                                                                    `ccaAAGGAGACATTCTGGATGTAACGTCCGTTGTCATTAATCCTGAGGCAGACTCTAAGGGCTTACAGCGATTCCTAGCGAAACGCTTTAAGTACCTTGCGGAACTCCACGATTGCGATTGGGTGTCTCGTTGTAAGCATGAAGGCGAGACAATGCGTGTATACTTTAAGGAGGTATAAGTTATGGGTAAGAAAGTTAAGAAGGCCGTGAAGAAAGTCACCAAGTCCGTTAAGAAAGTCGTTAAGGAAGGGGCTCGTCCGGTTAAACAGGTTGCTGGCGGTCTAGCTGGTCTGGCtggtgg                                                                                                                                                                                                                                                <  1:809243/300‑1 (MQ=255)`  
                                                                     `caAAGGAGACATTCTGGATGTAACGTCCGTTGTCATTAATCCTGAGGCAGACTCTAAGGGCTTACAGCGATTCCTAGCGAAACGCTTTAAGTACCTTGCGGAACTCCACGATTGCGATTGGGTGTCTCGTTGTAAGCATGAAGGCGAGACAATGCGTGTATACTTTAAGGAGGTATAAGTTATGGGTAAGAAAGTTAAGAAGGCCGTGAAGAAAGTCACCAAGTCCGTTAAGAAAGTCGTTAAGGAAGGGGCTAGTCCGGTTAAACAGGTTGCTGGCGGTCTAGCTGGTCTGGCTggtggt                                                                                                                                                                                                                                               >  1:537026/1‑301 (MQ=255)`  
                                                                     `aagagacagcATTCTGGATGTAACGTCCGTTGTCATTAATCCTGAGGAAGACTCTAAGGGCTTACAGCGATTCCTAGCGAAACGCTTTAAGTACCTTGCGGACCTCCACGATTGCGATTGGGTGTCTCGTTGTAAGCATGAAGGCGAGACAATGCGTGTATACTTTAAGGAGGTATAAGTTATGGGTAAGAAAGTTAAGAAGGCCGTGAAGAAAGTCACCAAGTCCGTTAAGAAAGTCGTTAAGGAAGGGGCTCGTCCGGTTAAACAGGTTGCTGGCGGTCTAGCTGGTATGGCTggtggt                                                                                                                                                                                                                                               <  1:206729/292‑1 (MQ=255)`  
                                                                         `ggAGACATTCTGGATGTAACGTCCGTTGTCATTAATCCTGAGGTAGACTCTAAGGGCTT‑CAGCGATTCCTAGCGAAACGCTTTAAGTACCTTGCGGAACTCCACGATTGCGATTGGGTGTCTCGTTGTAAGCATGAAGGCGAGACAATGCGTGTATACTTTAAGGAGGTATAAGTTATGGGTAAGAAAGTTAAGAAGGCCGTGAAGAAAGTCACCAAGTCCGTTAAGAAAGTCGTTAAGGAAGGGGCTCGTCCGGTTAAACAGGTTGCTGGCGGTCTAGCCTGTctcttatacacatctcc                                                                                                                                                                                                                                          >  1:474773/1‑286 (MQ=255)`  
                                                                            `gaCATTCTGGATGTAACGTCCGTTGTCATTAATCCTGAGGCAGACTCTAAGGGCTTACAGCGATTCCTAGCGAAACGCTTTAAGTACCTTGCGGAACTCCACGATTGCGATTGGGTGTCTCGTTGTAAGCATGAAGGCGAGACAATGCGTGTATACTTTAAGGAGGTATAAGTTATGGGTAAGAAAGTTAAGAAGGCCGTGAAGAAAGTCACCAAGTCCGTTAAGAAAGTCGTTAAGGAAGGGGCTCGTCCGGTTAAACAGGTTGCTGGCGGTCTAGCTGGTCTGGCTGGTGGTACTGGTg                                                                                                                                                                                                                                        >  1:431328/1‑301 (MQ=255)`  
                                                                                 `tCTGGATGTAACGTCCGTTGTCATTAATCCTGAGGCAGACTCTAAGGGCTTACAGCGATTCCTAGCGAAACGCTTTAAGTACCTTGCGGAACTCCACGATTGCGATTGGGTGTCTCGTTGTAAGCATGAAGGCGAGACAATGCGTGTATACTTTAAGGAGGTATAAGTTATGGGTAAGAAAGTTAAGAAGGCCGTGAAGAAAGTCACCAAGTCCGTTAAGAAAGTCGTTAAGGAAGGGGCTCGTCCGGTTAAACAGGTTGCTGGCGGTCTAGCTGGTCTGGCTGGTGGTACTGGTGAAGca                                                                                                                                                                                                                                   >  1:630746/1‑301 (MQ=255)`  
                                                                                       `tGTAACGTCCGTTGTCATTAATCCTGAGGCAGACTCTAAGGGCTTACAGCGATTCCTAGCGAAACGCTTTAAGTACCTTGCGGAACTCCACGATTGCGATTGGGTGTCTCGTTGTAAGCATGAAGGCGAGACAATGCGTGTATACTTTAAGGAGGTATAAGTTATGGGTAAGAAAGTTAAGAAGGCCGTGAAGAAAGTCACCAAGTCCGTTAAGAAAGTCGTTAAGGAAGGGGCTAGTCCGGTTAAACAGGTTGCTGGCGGTCTAGCTGGTCTGGCTGGTGGTACTGGTGAAGCACAGAtg                                                                                                                                                                                                                             <  1:487804/301‑1 (MQ=255)`  
                                                                                        `gTAACGTCCGTTGTCATTAATCCTGAGGCAGACTCTAAGGGCTTACAGCGATTCCTAGCGAAACGCTTTAAGTACCTTGCGGAACTCCACGATTGCGATTGGGTGTCTCGTTGTAAGCATGAAGGCGAGACAATGCGTGTATACTTTAAGGAGGTATAAGTTATGGGTAAGAAAGTTAAGAAGGCCGTGAAGAAAGTCACCAAGTCCGTTAAGAAAGTCGTTAAGGAAGGGGCTCGTCCGGTTAAACAGGTTGCTGGCGGTCTAGCTGGTCTGGCTGGTGGTACTGTTGAAGCACAGAtgg                                                                                                                                                                                                                            >  1:638212/1‑301 (MQ=255)`  
                                                                                            `ggggctcggagatgtgtataagagacagctctAAGGGCTTACAGCGATTCCTAGCGAAACGCTTTAAGTACCTTGCGGAACTCCACGATTGCGATTGGGTGTCTCGTTGTAAGCATGAAGGCGAGACAATGCGTGTATACTTTAAGGAGGTATAAGTTATGGGTAAGAAAGTTAAGAAGGCCGTGAAGAAAGTCACCAAGTCCGTTAAGAAAGTCGTTAAGGAAGGGGCTCGTCCGGTTAAACAGGTTGCTGGCGGTCTAGCTGGTCTGGCTGGTGGTACTGGTGAAGCACAGATGGTGGa                                                                                                                                                                                                                        <  1:239011/273‑1 (MQ=255)`  
                                                                                               `ccGTTGTCATTAATCCTGAGGCAGACTCTAAGGGCTTACAGCGATTCCTAGCGAAACGCTTTAAGTACCTTGCGGAACTCCACGATTGCGATTGGGTGTCTCGTTGTAAGCATGAAGGCGAGACAATGCGTGTATACTTTAAGGAGGTATAAGTTATGGTTAAGAAAGTTAAGAAGGCCGTGAAGAAAGTCACCAAGTCCGTTAAGAAAGTCGTTAAGGAAGGGGCTCGTCCGGTTAAACAGGTTGCTGGCGGTCTGGCTGGTCTGGCTGGTGGTACTGGTGAAGCACAGATGGTGGACGt                                                                                                                                                                                                                     >  1:669094/1‑301 (MQ=255)`  
                                                                                                    `gTCATTAATCCTGAGGCAGACTCTAAGGGCTTACAGCGATTCCTAGCGAAACGCTTTAAGTACCTTGCGGAACTCCACGATTGCGATTGGGTGTCTCGTTGTAAGCATGAAGGCGAGACAATGCGTGTATACTTTAAGGAGGTATAAGTTATGGGTAAGAAAGTTAAGAAGGCCGTGAAGAAAGTCACCAAGTCCGTTAAGAAAGTCGTTAAGGAAGGGGCTCGTCCGGTTAAACAGGTTGCTGGCGGTCTAGCTGGTCTGGCTGGTGGTACTGGTGAAGCACAGATGGTGGAAGTACcac                                                                                                                                                                                                                >  1:757593/1‑301 (MQ=255)`  
                                                                                                      `cATTAATCCTGAGGCAGACTCTAAGGGCTTACAGCGATTCCTAGCGAAACGCTTTAAGTACCTTGCGGAACTCCACGATTGCGATTGGGTGTCTCGTTGTAAGCATGAAGGCGAGACAATGCGTGTATACTTTAAGGAGGTATAAGTTATGGGTAAGTAAGTTAAGAAGGCCGTGAAGAAAGTCACCAAGTCCGTTAAGAAAGTCGTTAAGGAAGGGGCTCGTCCGGTTAAACAGGTTGCTGGCGGTCTAGCTGGTCTGGCTGGTGGTACTGGTGAAGCACAGATGGTGGAAGTACCACaa                                                                                                                                                                                                              >  1:786203/1‑301 (MQ=255)`  
                                                                                                       `aTTAATCCGGAGGAAGACTCGAAGGGCTTACAGCGATTCCTAGCGAAACGCTTTAAGTACCTTGCGGAACTCCACGATTGCGATTGGGTGTCTCGTTGTAAGCATGAAGGCGAGACAATGCGTGTATACTTTAAGGAGGTATAAGTTATGGGTAAGAAAGTTAAGAAGGCCGTGAAGAAAGTCACCAAGTCCGTTAAGAAAGTCGTTAAGGAAGGGGCTCGTCCGGTTAAACAGGTTGCTGGCGGTCTAGCTGGTCTGGCTGGTGGTACTGGTGAAGCACAGATGGTGGAAGTACCACAAg                                                                                                                                                                                                             <  1:251545/301‑1 (MQ=255)`  
                                                                                                                      `gACTCTAAGGGCTTACAGCGATTCCTAGCGAAACGCTTTAAGTACCTTGCGGAACTCCACGATTGCGATTGGGTGTCTCGTTGTAAGCATGAAGGCGAGACAATGCGTGTATACTTTAATGAGGTATAAGTTATGGGTAAGAAAGTTAAGAAGGCCGTGAAGAAAGTCACCAAGTCCGTTAAGAAAGTCGTTAAGGAAGGGGCTCGTCCGGTTAAACAGGTTGCTGGCGGTCTAGCTGGTCTGGCTGGTGGTACTGGTGAAGCACAGATGGTGGAAGTACcacccgtctcttatacacatc                                                                                                                                                                                              >  1:558446/1‑283 (MQ=255)`  
                                                                                                                      `gACTCTAAGGGCTTACAGCGATTCCTAGCGAAACGCTTTAAGTACCTTGCGGAACTCCACGATTGCGATTGGGTGTCTCGTTGTAAGCATGAAGGCGAGACAATGCGTGTATACTTTAAGGAGGTATAAGTTATGGGTAAGAAAGTTAAGAAGGCCGTGAAGAAAGTCACCAAGTCCGTTAAGAAAGTCGTTAAGGAAGGGGCTCGTCCGGTTAAACAGGTTGCTGGCGGTCTAGCTGGTCTGGCTGGTGGTACTGGTGAAGCACAGATGGTGGAAGTACCACAAGCTGCCGCACAGAttg                                                                                                                                                                                              >  1:108775/1‑301 (MQ=255)`  
                                                                                                                        `ctctAAGGGCTTACAGCTATTCCTAGCGAAACGCTTTAAGTACCTTGCGGAACTCCACGATTGCGATTGGGTGTCTCGTTGTAAGCATGAAGGCGAGACAATGCGTGTATACTTTAAGGAGGTATAAGTTATGGGTAAGAAAGTTAAGAAGGCCGTGAAGAAAGTCACCAAGTCCGTTAAGAAAGTCGATAAGGAAGGGGCTCGTCCGGTTAAACAGGTTGCTGGCGGTCTAGCTGGTCTGGCTGGTGGTACTGGTGAAGCACAGATGGTGGAGGTACCACAAGCTGCCGCACAGAttgtt                                                                                                                                                                                            >  1:676080/1‑301 (MQ=255)`  
                                                                                                                        `ctctAAGGGCTTACAGCGATTCCTAGCGAAACGCTTTAAGTACCTTGCGGAACTCCACGATTGCGATTGGGTGTCTCGTTGTAAGCATGAAGGCGAGACAATGCGTGTATACTTTAAGGAGGTATAAGTTATGGGTAAGAAAGTTAAGAAGGCCGTGAAGAAAGTCACCAAGTCCGTTAAGAAAGTCGTTAAGGAAGGGGCTCGTCCGGTTAAACAGGTTGCTGGCGGTCTAGCTGGTCTGGCTGGTGGTACTGGTGAAGCACATATGGTGGAAGTACCACAAGCTTCCGCACAGAttgtt                                                                                                                                                                                            >  1:191946/1‑301 (MQ=255)`  
                                                                                                                        `ctctAAGGGCTTACAGCGATTCCTAGCGAAACGCTTTAAGTACCTTGCGGAACTCCACGATTGCGATTGGGTGTCTCGTTGTAAGCATGAAGGCGAGACAATGCGTGTATACTTTAAGGAGGTATAAGTTATGGGTAAGAAAGTTAAGAAGGCCGTGAAGAAAGTCACCAAGTCCGTTAAGAAAGTCGTTAAGGAAGGGGCTCGTCCGGTTAAACAGGTTGCTGGCGGTCTAGCTGGTCTGGCTGGTGGTACTGGTGAAGCACAGATGGTGGAAGTACCACAAGCTGCCacacctgtctct                                                                                                                                                                                            >  1:601480/1‑293 (MQ=255)`  
                                                                                                                          `ctAAGGGCTTACAGCGATTCCTAGCGAAACGCTTTAAGTACCTTGCGGAACTCCACGATTGCGATTGGGTGTCTCGTTGTAAGCATGAAGGCGAGACAATGCGTGTATACTTTAAGGAGGTATAAGTTATGGGTAAGAAAGTTAAGAAGGCCGTGAAGAAAGTCACCAAGTCCGTTAAGAAAGTCGTTAAGGAAGGGGCTCGTCCGGTTAAACAGGTTGCTGGCGGTCTAGCTGGTCTGGCTGGTGGTACTGGTGAAGCACAGATGGTGGAAGTACCACAAGCTGCCGCACAGATTGTTGa                                                                                                                                                                                          <  1:579439/301‑1 (MQ=255)`  
                                                                                                                          `ctAAGGGCTTACAGCGATTCCTAGCGAAACGCTTTAAGTACCTTGCGGAACTCCACGATTGAGATTGGGTGTCTCGTTGTAAGCATGAAGGCGATACAATGCGTGTATACTTTAAGGAGGTATAAGTTATGGGTAAGAAAGTTAAGAAGGCCGTGAAGAAAGTCACCAAGTCCGTTAAGAAAGTCGTTAAGGAAGGGGCTCGTCCGGTTAAACAGGTTGCTGGCGGTCTAGCTGGTCTGGCTGGTGGTACTGGTGAAGCACAGATGGTGGAAGTACCACAAGCTGCCGCACAGATTGTTGa                                                                                                                                                                                          <  1:92566/301‑1 (MQ=255)`  
                                                                                                                          `ctAAGGGCTTACAGCGATTCCTAGCGAAACGCTTTAAGGACCTTGCGGAACTCCACGATTGCGATTGGGTGTCTCGTTGTAAGCATGAAGGCGAGACAATGCGTGTATACTTTAAGGAGGTATAAGTTATGGGTAAGAAAGTTAAGAAGGCCGTGAAGAAAGTCACCAAGTCCGTTAAGAAAGTCGTTAAGGAAGGGGTTCGTCCGGTTAAACAGGTTGCTGGCGGTCTAGCTGGTCTGGCTGGTGGTACTGGTGAAGCACAGATGGTGGAAGTACCACAAGCTGCCGCACAGATTGTTGa                                                                                                                                                                                          <  1:545094/301‑1 (MQ=255)`  
                                                                                                                            `caGGGCTTACAGCGATTCCTAGCGAAACGCTTTAAGTACCTTGCGGAACTCCACGATTGCGATTGGGTGTCTCGTTGTAAGCATGAAGGCGAGACAATGCGTGTATACTTTAAGGAGATATAAGTTATGGGTAAGAAAGTTAAGAAGGCCGTGAAGAAAGTCACCAAGTCCGTTAAGAAAGTCGTTAAGGAAGGGGCTCGTCCGGTTAAACAGGTTGCTGGCGGTCTAGCTGGTCTGGCTGGTGGTACTGGTGAAGCACAGATGGTGGAAGTACCACAAGCTGCCGCACAGATTGTTGACg                                                                                                                                                                                        <  1:313717/300‑1 (MQ=255)`  
                                                                                                                                `gCTTACAGCGATTCCTAGCGAAACGCTTTAAGTACCTTGCGGAACTCCACGATTGCGATTGGGTGTCTCGTTGTAAGCATGAAGGCGAGACAATGCGTGTATACTTTAAGGAGGTATAAGTTATGGGTAAGAAAGTTAAGAAGGCCGTGAAGAAAGTCACCAAGTCCGTTAAGAAAGTCGTTAAGGAAGGGGCTCGTCCGGTTAAACAGGTTGCTGGCGGTCTAGCTGGTCTGGCTGGTGGTACTGGTGAAGCACAGATGGTGGAAGTACCACAAGCTGCCGCACAGATTGTTGACGTAcc                                                                                                                                                                                    <  1:16215/301‑1 (MQ=255)`  
                                                                                                                                                  `cGAAACGCTTTAAGTACCTTGCGGAACTCCACGAGTGCGATTGGGTGTCTCGTTGTAAGCATGAAGGCGAGACAATGCGTGTATACTTTAAGGAGGTATAAGTTATGGGTAAGAAAGTTAAGAAGGCCGTGAAGAAAGTCACCAAGTCCGTTAAGAAAGTCGTTAAGGAAGGGGCTCGTCCGGTTAAACAGGTTGCTGGCAGTCTAGCTGGTCTGGCTGGTGGTACTGGTGAAGCACAGATGGTGGAAGTACCACAAGCTGCCGCACAGATTGTTGACGTACCTGAGAAAGAGGTTTCCAc                                                                                                                                                                  <  1:80249/301‑1 (MQ=255)`  
                                                                                                                                                                   `cTTGCGGAACTCCACGATTGCGATTGGGTGTCTCGTTGTACGCATGAAGGCGAGACAATGCGTGTATACTTTAAGGAGGTCTAAGTTATGGGTAAGAAAGTTAAGAAGGCCGTGAAGAAAGTCACCAAGTCCGTTAAGAAAGTCGTTAAGGAAGGGGCTCGTCCGGTTAAACAGGTTGCTGGCGGTCTAGCTGGTATGGCTGGTGGTACTGGTGAAGCACAGATGGTGGTAGTACCACAAGCTGCCGCACAGATTGTTGACGTAGCTGAGAAAGAGGTTGTCACTGAGGACGCAGCacaga                                                                                                                                                 >  1:513282/1‑301 (MQ=255)`  
                                                                                                                                                                    `ttGCGGAACTCCAAGATTGCGATTGTGTGTCTCATTGTAAGCATGAAGGCGAGAAAAGTCGTGTATACTTTAAGGAGGGATAAGTTATGGGTCAGAAAGTTAAGAAGGCCGTGAAGAAAGTCACCAATTCCGTTAAGAAAGTCGTTAAGGAAGGGGCTCGTCCGGTTAAACAGGTTGCTGGCGGTCTAGCTGGTCTGGCTGGTGGTACTGGTGAAGCACAGATGGTGGAAGTACCACAAGCTGCCGCACAGATTGTTGACTTACCTGAGAAAGAGGTTTCCACTGAGGACGAAGCacagac                                                                                                                                                <  1:764478/301‑1 (MQ=255)`  
                                                                                                                                                                    `agtagctgcaccaggtttgcgattgGGTGTCTCGTTGTAATCATGAAGGCGAGACAATGCGTGTAAAGTTTAAGGAGGTATAAGTTATGGGTAAGGAAGTGATGAAGGCCGTGAAGAAAGTCACCAAGTCCGTTAAGAAAGTCGTTAAGGAAGGGGCTTGTCCGGTTAAGCAGGTTGCTGTCGGTCTAGCTGGTCTGGCTGGTGGTACTGGTGAAGCACAGATGGTGGAAGTACCACAAGCTGCCGCACAGATTGTTGACGTACCTGAGAAAGAGGTTTCCACTGAGGACGAAGCacagac                                                                                                                                                <  1:306746/285‑1 (MQ=255)`  
                                                                                                                                                                        `ggatcgtcACGATTGAGATTGGGTTTCTCGTTGTAAGCATGAAGGCGAGACAATGCGTTTATACTTTAAGGAGGTAAAAGGTATGGGTAAGAAAGTTAAGAAGGCCGTGAAGAAAGTCACCAAGTCCGTTAAGAAAGTCGTTAAGGACGGGGCTCGTCCGGTTAAACAGGTTGCTGGTGGTCTAGCTGGTCTGGCTGGTGGTACTGGTGAAGCACAGATGGTGGAAGTACCACAAGCTGCCGCACAGATTGTTGACGTACCTGAGAAAGAGGTTTCCACTGAGGACGAAGCACAGACAGaa                                                                                                                                            <  1:100102/294‑1 (MQ=255)`  
                                                                                                                                                                         `gAACTCCACGATTGCGATTGGGTGTCTCGTTGTAAGCATGAAGGCGAGACAATGCGTGTATACTTTGAGGAGGTATAAGTTATGGGTAAGAAAGTTAAGAAGGCCGTGAAGAAAGTCACCAAGTCCGTTAAGAAAGTCGTTAAGGAAGGGGCTCGTCCGGTTAAACAGGTTGCTGGCGGTCTAGCTGGTCTGGCTGGTGGTACTGGTGAAGCACAGATGGTGGAAGTACCACAAGCTGCCGCACAGATTGTTGACGTACCTGAGAAAGAGGTTTCCACTGAGGACGAAGCACAGACAGaaa                                                                                                                                           >  1:33532/1‑301 (MQ=255)`  
                                                                                                                                                                         `gAACTCCACGATTGCGATTGGGTGTCTCGTTGTAAGCATGAAGGCGAGACAATGCGTGTATACTTTAAGGAGGTATAAGTTATGGGTAAGAAAGTTAAGAAGGCCGTGAAGAAAGTCACCAAGTCCGTTAAGAAAGTCGTTAAGGAAGGGGCTCGTCCGGTTAAACAGGTTGCTGGCGGTCTAGCTGGTCTGGCTGGTGGTACTGGTGAAGCACAGATGGTGGAAGTACCACAAGCTGCCGCACAGATTGTTGACGTACCTGAGAAAGAggctgtctcttatacacatctccgagcccacg                                                                                                                                           >  1:440252/1‑271 (MQ=255)`  
                                                                                                                                                                                         `attgGGTGTCTCGTTGTAAGCATGAAGGCGAGACAATGCGTGTATACTTTAAGGAGGTATAAGTTATGGGTAAGAAAGTTAAGAAGGCCGTGAAGAAAGTCACCAAGTCCGTTAAGAAAGTCGTTAAGGAAGGGGCTCGTCCGGTTAAACAGGTTGCTGGCGGTCTAGCTGGTCTGGCTGGTGGTACTGGTGAAGCACAGATGGTGGAAGTACCACAAGCTGCCGCACAGATTGTTGACGTACCTGAGAAAGAGGTTTCCACTGAGGACGAAGCACAGACAGAAAGAGGACGCAAGAAAGc                                                                                                                           >  1:453515/1‑301 (MQ=255)`  
                                                                                                                                                                                          `ttgGGTTTCTCTATGTAAGCATGAAGGCGATACAATGCGTGTATATTTTAAGGAGGTATAAGTTATGGGTAAGAAAGTTAAGACGGCCGTGAACAAAGTCACCAAGTCCGTTAAGAAAGTCGTTAAGGAAGGGGCTCGTCCGGTTAATCAGGTTTCTGGCGGTCTAGCTGGTCTGGCTGGTGGTACTGGTGAAGCACAGATTGTGGAAGTACCACAAGCTGCCGCACATATTGTTGACGTACCTGAGAAAGACGTTTCCACTGAGGACGAAGCACAGACAGAAAGCGGACGCAAGAAAGCt                                                                                                                          <  1:722048/301‑1 (MQ=255)`  
                                                                                                                                                                                                          `aggCATGAAGGGGAGACAATGCGTGTAGACTTTAAGGAGGTATAAGTTATGGGTAAGAAAGTTAAGAAGGCCGTGAAGAAAGTCACCAAGTC‑‑‑‑‑‑‑‑‑‑‑‑CGTTAAGGAAGGGGCTCGTCCGGTTAAACAGGTTGCTGGCGGTCTAGCTGGTCTGGCTGGTGGTACTGGTGAAGCACAGATGGTGGAAGTACCACAAGCTGCCGCACAGATTGTTGACGTACCTGAGAAAGAGGGTTCCACTGAGGACGAAGCACAGACAGAAAGCGGACGCAAGAAAGCTCGTGCTGGCGGTAAGAAATCCTTGAgcg                                                                                              <  1:565360/299‑3 (MQ=255)`  
                                                                                                                                                                                                            `gCATGAAGGCGAGACAATGCGTGTATACTTTAAGGAGGTATAAGTTATGGGTAAGAAAGTTAAGAATGCCGTGAAGAAAGTCACCAAGTCCGTTAAGAAAGTCGTTAAGGAAGGGGCTCGTCCGGCTAAACAGGATGCTGGAGGTCTAGCTGGTCTGGCTGGTGGTACTGGTGAAGCACAGATGGTGGACGTAACACAAGCTGCCGCACAGATTGTTGACGTACCTGAGAAAGAGGTTTCCACTGAGGGCGACGCACAGACAGCACGCGGACGCAAGAAAGCTCGAGCAGGCGGTAAGgaa                                                                                                        >  1:12807/1‑301 (MQ=255)`  
                                                                                                                                                                                                                  `ttgtataagagacagtgtATACTTTAAGGAGGTATAAGTTATGGGTAAGAAAGTTAAGAAGGCCGTGAAGAAAGTCACCAAGTCCGTTAAGAAAGTCGTTAAGGAAGGGGCTCGTCCGGTTAAACAGGTTGCTGGCGGTCTAGCTGGTCTGGCTGGTGGTACTGGTGAAGCACAGATGGTGGAAGTACCACAAGCTGCCGCACAGATTGTTGACGTACCTGAGAAAGAGGTTTCCACTGAGGACGAAGCACAGACAGAAAGCGGACGCAAGAAAGCTCGTGCTGGCGGTAAGAAATCCTTg                                                                                                  <  1:586898/287‑1 (MQ=255)`  
                                                                                                                                                                                                                   `ggCGAGACAATGCGTGTATACTTTAAGGAGGTATAAGTTATGGGTAAGAAAGTTAAGAAGGCCGTGAAGAAAGTCACCAAGTCCGTTAAGAAAGTCGTTAAGGAAGGGGCTCGTCCGGTTAAACAGGTTGCTGGCGGTCTAGCTGGTCTGGCTGGTGGTACTGGTGAAGCACAGATGGTGGAAGTACCACAAGCTGCCGCACAGATTGTTGACGTACCTGAGAAAGAGGTTTCCACTGAGGACGAAGCACAGACAGAAAGCGGACGCAAGAAAGCTCATGCTGGCGGTAAGAAATCCTTgc                                                                                                 >  1:421523/1‑300 (MQ=255)`  
                                                                                                                                                                                                                   `ggCGAGACAATGCGTGTATACTTTAAGGAGGTATAAGTTATGGGTAAGAAAGTTAAGAAGGCCGTGAAGAAAGTCACCAAGTC‑‑‑‑‑‑‑‑‑‑‑‑CGTTAAGGAAGGGGCTCGTCCGGTTAAACAGGTTGCTGGCGGTCTAGCTGGTATGGCTGGTGGTACTGGTGAAGCACAGATGGTGGAAGTACCACAAGCTGCCGCACAGATTGTTGACGTACCTGAGAAAGAGGTTTCCACTGAGGACGAAGCACAGACAGAAAGCGGACGCAAGAAAGCTCGTGCTGGCGGTAAGAAATCCTTGAGTGTAGCCCGTa                                                                                     >  1:264830/1‑301 (MQ=255)`  
                                                                                                                                                                                                                    `gCGAGACAATGCGTGTATACTTTAAGGAGGTATAAGTTATGGGTAAGAAAGTTAAGAAGGCCGTGAAGAAAGTCACCAAGTCCGTTAAGAAAGTCGTTAAGGAAGGGGCTCGTCCGGTTAAACAGGTTGCTGGCGGTCTAGCTGGTCTGGCTGGTGGTACTGGTGAAGCACAGATGGTGGAAGTACCACAAGCTGCCGCACAGATTGTTGACGTACCTGAGAAAGAGGTTTCCACTGAGGACGAAGCACAGACAGAAAGCGGACGCAAGAAAGCTCGTGCTGGCGGTAAGAAATCCTTGAg                                                                                                <  1:73156/301‑1 (MQ=255)`  
                                                                                                                                                                                                                      `gagaCAATGCGTGTATACTTTAAGGAGGTATAAGTTATGGGTAAGAAAGTTAAGAAGGCCGTGAAGAAAGTCACCAAGTCCGTTAAGAAAGTCGTTAAGGAAGGGGCTCGTCCGGTTAAACAGGTTGCTGGCGGTCTAGCTGGTCTGGCTGGTGGTACTGGTGAAGCACAGATGGTGGAAGTACCACAAGCTGCCGCACAGATTGTTGACGTACCTGAGAAAGAGGTTTCCACTGAGGACGAAGCACAGACAGAAAGCGGACGCAAGAAAGCTCGTGCTGGCGGTAAGAAATCCTTGAgtg                                                                                              <  1:623419/301‑1 (MQ=255)`  
                                                                                                                                                                                                                          `aatagtggacagagtttAAGGAGGTAAAAGTTATGGGTAAGAAAGTTAAGAAGGCCGTGAAGAAAGTCACCAAGTC‑‑‑‑‑‑‑‑‑‑‑‑CGTTAAGGAAGGGGCTCGTCCGGTTAAACAGGTTTCTGGCGGTCTAGCTGGTCTGGCTGGTGGTACTGGTGAAGCACAGATGGTGGAAGTACCACAAGCTGCCGCACAGATTGTTGACGTACCTGAGAAAGAGGTTTCCACTGAGGACGAAGCACAGACAGAAAGCGGACGCAAGAAAGCTCGTGCTGGCGGTAAGAAATCCTTGAGTGTAGCCCGTAGCTCCgg                                                                              <  1:521856/287‑1 (MQ=255)`  
                                                                                                                                                                                                                           `aaagggttgataCTTTAAGGAGGTATAAGTTATGGGTAAGAAAGTTAAGAAGGCCGTGAAGAAAGTCACCAAGTCCGTTAATAAAGTCGTTAAGGAAGGGGCTCGTCCGGTTAAACAGGTTGCTGGCGGTCTAGCTGGTCTGGCTGGTGGTACTGGTGAAGCACAGATGGTGGAAGTACCACAAGCTGCCGCACAGATTGTTGACGTACCTGAGAAAGAGGTTTCCACTGAGGACGAAGCACAGACAGAAAGCGGACGCAAGAAAGCTCGTGCTGGCGGTAAGAAATCCTTGAGTGTAGcc                                                                                         <  1:354023/292‑1 (MQ=255)`  
                                                                                                                                                                                                                             `tGCGTGTATACTTTAAGGAGGTATAAGTTATGGGTAAGAAAGTTAAGAAGGCCGTGAAGAAAGTCACCAAGTCCGTTAAGAAAGTCGTTAAGGAAGGGGCTCGTCCGGTTAAACAGGTTGCTGGCGGTCTAGCTGGTCTGGCTGGTGGTACTGGTGAAGCACAGATGGTGGAAGTACCACAAGCTGCCGCACAGATTGTTGACGTACCTGAGAAAGAGGTTTCCACTGAGGACGAAGCACAGACAGAAAGCGGACGCAAGAAAGCTCGTGCTGGCggctgtctcttatacacatctccgac                                                                                       >  1:551631/1‑277 (MQ=255)`  
                                                                                                                                                                                                                              `gCGTGTATACTTTCAGGAGGTATAAGTTATGGGTAAGAAAGTTAAGAAGGCCGTGAAGAAAGTCACCAAGTCCGTTAAGAAAGTCGTTAAGGAAGGGGCTCGTCCGGTTAAACAGGTTGCTGGCGGTCTAGCTGGTCTGGCTGGTGGTACTGGTGAAGCACAGATGGTGGAAGTACCACAAGCTGCCGCACAGATTGTTGACGTACCTGAGAAAGAGGTTTCCACTGAGGACGAAGCACAGACAGAAAGCGGACGCAAGAAAGCTCGTGCTGGCGGTAAGaactgtctcttatacacatct                                                                                      >  1:392281/1‑282 (MQ=255)`  
                                                                                                                                                                                                                              `gCGTGTATACTTTAAGGAGGTATAAGTTATGGGTAAGAAAGTTAAGAAGGCCGTGAAGAAAGTCACCAAGTCCGTTAAGAAAGTCGTTAAGTAAGGGGCTCGTCCGGTTAAACAGGTTGCTGGCGGTCTAGCTGGTCTGGCTGGTGGTACTGGTGAAGCACAGATGGTGGAAGTACCACAAGCTGCCGCACAGATTGTTGACGTACCTGAGAAAGAGGTTTCCACTGAGGACGAAGCACAGACAGAAAGCGGACGCAAGAAAGCTCGTGCTGGCGGTAAGAAATCCTTgctgtctcttata                                                                                      >  1:39945/1‑289 (MQ=255)`  
                                                                                                                                                                                                                              `gCGTGTATACTTTAAGGAGGTATAAGTTATGGGTAAGAAAGTTAAGAAGGCCGTGAAGAAAGTCACCAAGTCCGTTAAGAAAGTCGTTAAGGAAGGGGCTCGTCCGGTTAAACAGGTTGCTGGCGGTCTAGCTGGTCTGGCTGGTGGTACTGGTGAAGCACAGATGGTGGAAGTACCACAAGCTGCCGCACAGATTGTTGACGTACCTGAGAAAGAGGTTTCCACTGAGGACGAAGCACAGACAGAAAGCGGACGCAAGAAAGCTCGTGCTGGCGGTAAGAAATCCTTGAgtgtctgtctc                                                                                      >  1:446179/1‑294 (MQ=255)`  
                                                                                                                                                                                                                              `gCGTGTATACTTTAAGGAGGTATAAGTTATGGGTAAGAAAGTTAAGAAGGCCGTGAAGAAAGTCACCAAGTCCGTTAAGAAAGTCGTTAAGGAAGGGGCTCGTCCGGTTAAACAGGTTGCTGGCGGTCTAGCTGGTCTGGCTGGTGGTACTGGTGAAGCACAGATGGTGGAAGTACCACAAGCTGCCGCACAGATTGTTGACGTACCTGAGAAAGAGGTTTCCACTGAGGACGAAGCACAGACAGAAAGCGGACGCAAGAAAGCTCGTGCTGGCGGTAAGAAATCCTTGAGTGTAGCCCGt                                                                                      >  1:57365/1‑301 (MQ=255)`  
                                                                                                                                                                                                                                 `tgtATACTTTAAGGAGGTATAAGTTATGGGTAAGAAAGTTAAGAAGGCCGTGAAGAAAGTCACCAAGTCCGTTAAGAAAGTCGTTAAGGAAGGGGCTCGTCCGGTTAAACAGGTTGCTGGCGGTCTAGCTGGTCTGGCTGGTGGTACTGGTGAAGCACAGATGGTGGAAGTACCACAAGCTGCCGCACAGATTGTTGACGTACCTGAGAAAGAGGTTTCCACTGAGGACGAAGCACAGACAGAAAGCGGACGCAAGAAAGCTCGTGCTGGCGGTAAGAAATCCTTGAGTGTAGCCCGTAGc                                                                                   >  1:794034/1‑301 (MQ=255)`  
                                                                                                                                                                                                                                      `gcTTTAAGTAGGTATAAGTTATGGGTAAGAAAGTTAAGAAGGCCGTGAAGAAAGTCACCAAGTCCGTTAAGAAAGTCGTTAAGGAAGGGGCTCGTCCGGTTAAACAGGTTGCTGGCGGTCTAGCTGGTCTGGCTGGTGGTACTGGTGAAGCACAGATGGTGGAAGTACCACAAGCTGCCGCACAGATTGTTGACGTACCTGAGAAAGAGGTTTCCACTGAGGACGAAGCACAGACAGAAAGCGGACGCAAGAAAGCTCGTGCTGGCGGTAAGAAATCCTTGAGTGTAGCCCGTAGCTCCgg                                                                              <  1:328205/300‑1 (MQ=255)`  
                                                                                                                                                                                                                                      `aCTTTAAGGATGTATAAGTTATGGGTAAGAAAGTTAAGAAGGCCGTGAAGAAAGTCACCAAGTCCGTTAAGAAAGTCGTTAAGGAAGGGGCTCGTCCGGTTAAACAGGTTGCTGGCGGTCTAGCTGGTCTGGCTGGTGGTACTGGTGAAGAACAGATGGTGGAAGTACCACAAGCTGCCGCACAGATTGTTGACGTACCTGAGAAAGAGGTTTCCACTGAGGACGACGCACAGACAGAAAGCGGACGCAAGAAAGCTCGTGCTGGCGGTAAGAAATCCTTGAGTGTAGCCAGTAGCCCCgg                                                                              >  1:655838/1‑301 (MQ=255)`  
                                                                                                                                                                                                                                      `aCTTTAAGGAGGTATAAGTTATGGGTAAGAAAGTTAAGAAGGCCGTGAAGAAATTCACCAAGTCCGTTAAGAAAGTCGTTAAGGAAGGGGCTCGTCCGGTTAAACAGGTTGCTGGCGGTCTAGCTGGTCTGGCTGGTGGTACTGGTGAAGCACAGATGGTGGAAGTACCACAAGCTGCCGCACAGATTGTTGACGTACCTGAGAAAGAGGTTTCCACTGAGGACGAAGCACAGACAGAAAGCGGACGCAAGAAAGCTCGTGCTGGCGGTAAGAAATCCTTGAGTGTAGCCCGTAGCTCCgg                                                                              >  1:133453/1‑301 (MQ=255)`  
                                                                                                                                                                                                                                       `cTTTAAGGAGGTATAAGTTATGGGTAAGAAAGTTAAGAAGGCCGTGAAGAAAGTCACCAAGTCCGTTAAGAAAGTCGTTAAGGAAGGGGCTCGTCCGGTTAAACAGGTTGCTGGCGGTCTAGCTGGTCTGGCTGGTGGTACTGGTGAAGCACAGATGGTGGAAGTACCACAAGCTGCCGCACAGATTGTTGACGTACCTGAGAAAGAGGTTTCCACTGAGGACGAAGCACAGACAGAAAGCGGACGCAAGAAAGCTCGTGCTGGCGGTAAGAAATCCTTGAGTGTAGCCCGTAGCTCCGGt                                                                             >  1:245086/1‑301 (MQ=255)`  
                                                                                                                                                                                                                                         `ttAAGGAGGTATAAGTTATGGGTAAGAAAGTTAAGAAGGCCGTGAAGAAAGTCACCAAGTCCGTTAAGAAAGTCGTTAAGGAAGGGGCTCGTCCGGTTAAACAGGTTGCTGGCGGTCTAGCTGGTCTGGCTGGTGGTACTGGTGAAGCACAGATGGTGGAAGTACCACAAGCTGCCGCACAGATTGTTGACGTACCTGAGAAAGAGGTTTCCACTGAGGACGAAGCACAGACAGAAAGCGGACGCAAGAAAGCTCGTGCTGGCGGTAAGAAATCCTTGAGTGTAGCCCGTAGCTCCAGTgg                                                                           <  1:141066/301‑1 (MQ=255)`  
                                                                                                                                                                                                                                         `gacaggaggTATAAGTTATGGGTAAGAAAGTTAAGAAGGCCGTGAAGAAAGTCCCCAAGTCCGTTAAGAAAGTCGTTAAGGAAGGGGCTCGTCCGGTTAAACAGGTTGCTGGCGGTCTAGCTGGTCTGGCTGGTGGTACTGGTGAAGCACAGATGGTGGAAGTACCACAAGCTGCCGCACAGATTGTTGACGTACCTGAGAAAGAGGTTTCCACTGAGGACGAAGCACAGACAGAAAGCGGACGCAAGAAAGCTCGTGCTGGCGGTAAGAAATCCTTGAGTGTAGCCCGTAGCTCCGGTgg                                                                           <  1:70644/298‑1 (MQ=255)`  
                                                                                                                                                                                                                                           `gacagggTATAAGTTATGGGTAAGAAAGTTAAGAAGGCCGTGAAGAAAGTCACCAAGTCCGTTAAGAAAGTCGTTAAGGAAGGGGCTCGTCCGGTTAAACAGGTTGCTGGCGGTCTAGCTGGTCTGGCTGGTGGTACTGGTGAAGCACAGATGGTGGAAGTACCACAAGCTGCCGCACAGATTGTTGACGTACCTGAGAAAGAGGTTTCCACTGAGGACGAAGCACAGACAGAAAGCGGACGCAAGAAAGCTCGTGCTGGCGGTAAGAAATCCTTGAGTGTAGCCCGTAGCTCCGGTGGCg                                                                         <  1:714718/296‑1 (MQ=255)`  
                                                                                                                                                                                                                                              `gaggTATAAGTTATGGGTAAGAAAGTTAAGAAGGCCGTGAAGAAAGTCACCAAGTCCGTTAAGAAAGTCGTTAAGGAAGGGGCTCGTCCGGTTAAACAGGTTGCTGGCGGTCTAGCTGGTCTGGCTGGTGGTACTGGTGAAGCACAGATGGTGGAAGTACCACAAGCTGCCGCACAGATTGTTGACGTACCTGAGAAAGAGGTTTCCACTGAGGACGAAGCACAGACAGAAAGCGGACGCAAGAAAGCTCGTGCTGGCGGTAAGAAATCCTTGAGTGTAGCCCGTAGCTCCGGTGGCGGTa                                                                      >  1:691446/1‑301 (MQ=255)`  
                                                                                                                                                                                                                                               `aggTATAAGTTATGGGTAAGAAAGTTAAGAAGGCCGTGAAGAAAGTCACCAAGTCCGTTAAGAAAGTCGTTAAGGAAGGGGCTCGTCCGGTTAAACAGGTTGCTGGCGGTCTAGCTGGTCTGGCTGGTGGTACTGGTGAAGCACAGATGGTGGAAGTACCACAAGCTGCCGCACAGATTGTTGACGTACCTGAGAAAGAGGTTTCCACTGAGGACGAAGCACAGACAGAAAGCGGACGCACGAAAGCTCGTGCTGGCGGTAAGAAATCCTTGAGTGTAGCCCGTAGCTCCGGTGGCGGTAt                                                                     >  1:272553/1‑301 (MQ=255)`  
                                                                                                                                                                                                                                               `aggTATAAGTTATGGGTAAGAAAGTTAAGAAGGCCGTGAAGAAAGTCACCAAGTCCGTTAAGAAAGTCGTTAAGGAAGGGGCTCGTCCGGTTAAACAGGTTGCTGGCGGTCTAGCTGGTCTGGCTGGTGGTACTGGTGAAGCACAGATGGTGGAAGTACCACAAGCTGCCGCACAGATTGTTGACGTACCTGAGAAAGAGGTTTCCACTGAGGACGAAGCACAGACAGAAAGCGGACGCAAGAAAGCTCGTGCTGGCGGTAAGAAATCCTTGAGTGTAGCCCGTAGcctgtctcttataca                                                                     >  1:481843/1‑287 (MQ=255)`  
                                                                                                                                                                                                                                                `ggTCTAAGTTATGGGTAAGAAAGTTAAGAAGGCCGTGAAGAAAGTCACCAAGTCCGTTAAGAAAGTCGTTAAGGAAGGGGCTCGTCCGGTTAAACAGGTTGCTGGCGGTCTAGCTGGTCTGGCTGGTGGTACTGGTGAAGCACAGATGGTGGAAGTACCACAAGCTGCCGCACAGATTGTTGACGTACCTGAGAAAGAGGTTTCCACTGAGGACGAAGCACAGACAGAAAGCGGACGCAAGAAAGCTCGTGCTGGCGGTAAGAAATCCTTGAGTGTAGCCCGTAGCTCCGGTGGCGGTATc                                                                    >  1:299125/1‑301 (MQ=255)`  
                                                                                                                                                                                                                                                `ggTATAAGTTATGGGTAAGAAAGTTAAGAAGGCCGTGAAGAAAGTCACCAAGTCCGTTAAGAAAGTCGTTAAGGAAGGGGCTCGTCCGGTTAAACAGGTTGCTGGCGGTCTAGCTGGTCTGGCTGGTGGTACTGGTGAAGCACAGATGGTGGAAGTACCACAAGCTGCCGCACAGATTGTTGACGTACCTGAGAAAGAGGTTTCCACTGAGGACGAAGCACAGACAGAAAGCGGACGCAAGAAAGCTCGTGCTGGCGGTAAGAAATCCTTGAgctgtctcttatacacatctccgagccca                                                                    >  1:662862/1‑273 (MQ=255)`  
                                                                                                                                                                                                                                                 `gTATAAGTTATGGGTAAGAAAGTTAAGAAGGCCGTGAAGAAAGTCACCAAGTCCGTTAAGAAAGTCGTTAAGGAAGGGGCTCGTCCGGTTAAACAGGTTGCTGGCGGTCTAGCTGGTCTGGCTGGTGGTACTGGTGAAGCACAGATGGTGGAAGTACCACAAGCTGCCGCACAGATTGTTGACGTACCTGAGAAAGAGGTTTCCACTGAGGACGAAGCACAGACAGAAAGCGGACGCAAGAAAGCTCGTGCTGGCGGTAAGAAATCCTTGAGTGTAGCCCGTAGCTCCGGTGGCGGTATCa                                                                   >  1:683363/1‑301 (MQ=255)`  
                                                                                                                                                                                                                                                 `gTATAAGTTATGGGTAAGAAAGTTAAGAAGGCCGTGAAGAAAGTCACCAAGTCCGTTAAGAAAGTCGTTAAGGAAGGGGCTCGTCCGGTTAAACAGGTTGCTGGCGGTCTAGCTGGTCTGGATGGTGGTACTGGTGAAGCACAGATGGTGGAAGTACCACAAGCTGCCGCACAGATTGTTGACGTACCTGAGAAAGAGGTTTCCACTGAGGACGAAGCACAGACAGAAAGCGGACGCAAGAAAGCTCGTGCTGGCGGTAAGAAATCCTTGAGTGTAGCCCGTAGCTCCGGTGGCGGTATCa                                                                   >  1:498069/1‑301 (MQ=255)`  
                                                                                                                                                                                                                                                             `ggTAAGAAAGTTAAGAAGGCCGTGAAGAAAGTCACCAAGTCCGTTAAGAAAGTCGTTAAGGAAGGGGCTCGTTCGGTTAAACAGGTTGCTGGCGGTCTAGCTGGTCTGGCTGGTGGTACTGGTGAAGCACAGATGGTGGAAGTACCACAAGCTGCCGCACAGATTGTTGACGTACCTGAGAAAGAGGTTTCCACTGAGGACGAAGCACAGACAGAAAGCGGACGCAAGAAAGCTCGTGCTGGCGGTAAGAAATCCTTGAGTGTAGCCCGTAGCTCCGGTGGCGGTATCAACATTTAATCag                                                       >  1:119729/1‑301 (MQ=255)`  
                                                                                                                                                                                                                                                              `gTAAGAAAGGTAAGAAGGCCGTGAAGAAAGTCACCAAGTCCGTTAAGAAAGTCGTTAAGGAAGGGGCTCGTCCGGTTAAACAGGTTGCTGGCGGTCTAGCTGGTCTGGCTGGTGGTACTGGTGAAGCACAGATGGTGGAAGTACCACAAGCTGCCGCACAGATTGTTGACGTACCTGAGAAATAGGTTTCCACTGAGGACGAAGCACAGACAGAAAGCGGACGCAAGAAAGCTCGTGCTGGCGGTAAGAAATCCTTGAGTGTAGCCCGTAGCTCCGGTGGCGGTATCAACATTTAATCagg                                                      <  1:504732/301‑1 (MQ=255)`  
                                                                                                                                                                                                                                                                     `aGTTAAGAAGGCCGTGAAGAAAGTCACCAAGTCCGTTAAGAAAGTCGTTAAGGAAGGGGCTCGTCCGGTTAAACAGGTTGCTGGCGGTCTAGCTGGTCTGGCTGGTGGTACTGGTGAAGCACAGATGGTGGAAGTACCACAAGCTGCCGCACAGATTGTTGACGTACCTGAGAAAGAGGTTTCCACTGAGGACGAAGCACAGACAGAAAGCGGACGCAAGAAAGCTCGTGCTTGCGGTAAGAAATCCTTGAGTGTAGCCCGTAGCTCCGGTGGCGGTATCAACATTTAATCAGGAGGTTAt                                               <  1:346283/301‑1 (MQ=255)`  
                                                                                                                                                                                                                                                                     `aGTTAAGAAGGCCGTGAAGAAAGTCACCAAGTCCGTTAAGAAAGTCGTTAAGGAAGGGGCTCGTCCGGTTAAACAGGTTGCTGGCGGTCTAGCTGGTCTGGCTGGTGGTACTGGTGAAGCACAGATGGTGGAAGGACCACAAGCTGCCGAACAGATTGTTGACGTACCTGAGAAAGAGGTTTCCACTGAGGACGAAGCACAGACAGAAAGCGGACGCAAGAAAGCTCGTGCTGGCGGTAAGAAATCCTTGAGTGTAGCCCGTAGCTCCGGTGGCGGTATCAACATTTAATCAGGAGGGTAt                                               <  1:171817/301‑1 (MQ=255)`  
                                                                                                                                                                                                                                                                      `gTTAAGAAGGCCGTGAAGAAAGTCACCAAGTCCGTTAAGAAAGTCGTTAAGGAAGGGGCTCGTCTGGTTAAACAGGTTGCTGGCGGTCTAGCTGGTCTGGCTGGTGGTACTGGTGAAGCACAGATGGTGGAAGTACCACAAGCTGCCGCACAGATTGTTGACGTACCTGAGAAAGAGGTTTCCACTGAGGACGAAGCACAGACAGAAAGCTGACGCAAGAAAGCTCGTGCTGGCGGTAAGAAATCCTTGAGTGTAGCCCGTAGCTCCTGTGGCGGTATCAACATTTAATCAGGAGGTTATc                                              >  1:178945/1‑301 (MQ=255)`  
                                                                                                                                                                                                                                                                      `gTTAAGAAGGCCGTGAAGAAAGTCACCAAGTCCGTTAAGAAAGTCGTTAAGGAAGGGGCTCGTCCGGTTAAACAGGTTGCTGGCGGTCTAGCTGGTCTGGCTGGTGGTACTGGTGAAGCTCAGATGGTGGAAGTACCACAAGCTGCCGCACAGATTGTTGACGTACCTGAGAAAGAGGTTTCCACTGAGGACGAAGCACAGACAGAAAGCGGACGCAAGAAAGCTCGTGCTGGCGGTAAGAAATCCTTGAGTGTAGCCCGTAGCTCCGGTGGCGGTATCAACATTTAATCAGGAGGTTAta                                              >  1:413474/1‑300 (MQ=255)`  
                                                                                                                                                                                                                                                                        `tAAGAAGGCCGTGAAGAAAGTCACCAAGTCCGTTAAGAAAGTCGTTAAGGAAGGGGCTCGTCCGGTTAAACAGGTTGCTGGCGGTCTAGCTGGTCTGGCTGGTGGTACTGGTGAAGCACAGATGGTGGAAGTACCACAAGCTGCCGCACAGATTGTTGACGTACCTGAGAAAGAGGTTTCCACTGAGGACGAAGCACAGACAGAAAGCGGACGCAAGAAAGCTCGTGCTGGCGGTAAGAAATCCTTGAGTGTAGCCCGTAGCTCCGGTGGCGGTATCAACATATAATCAGGAGGTTATCGt                                            <  1:816651/301‑1 (MQ=255)`  
                                                                                                                                                                                                                                                                               `gCCGTGAAGAAAGTCACCAAGTCCGTTAAGAAAGTCGTTAAGGAAGGGGCTCGTCCGGTTAAACAGGTTGCTGGCGGTCTAGCTGGTCTGACTGGTGGTACTGGTGAAGCACAGATGGTGGAAGTACCACAAGCTGCCGCACAGATTGTTGACGTACCTGAGAAAGAGGTTTCCACTGAGGACGAAGCACAGACAGAAAGCGGACGCAAGAAAGCTCGTGCTGGCGGTAAGAAATCCTTGAGTGTAGCCCGTAGCTCCGGTGGCGGTATCAACATTTAATCAGGAGGTTATCGTGGAAGAc                                     >  1:36839/1‑301 (MQ=255)`  
                                                                                                                                                                                                                                                                                 `cGTGAATAAAGTCACCAAGTCCGTTAAGAAAGTCGTTAAGGCAGGGGCTCGTCCGGTTAAACAGGTTGCTGGCGGTCTAGCTGGTCTGGCTGGTGGTACTGGTGAAGCACAGATGGTGGAAGTACCACAAGCTGCCGTACAGATTGTTGACGTACCTGAGAAAGAGGTTTCCACTGAGGACGAAGCACAGACAGAAAGCGGACGCAAGAAAGCTCGTGCTGGCGGTAAGAAATCCTTGAGTGTAGCCCGTAGCTCCGGTGGCGGTATCAACATTTAATCAGGAGGTTATCGTTGAAGACtc                                   >  1:750030/1‑300 (MQ=255)`  
                                                                                                                                                                                                                                                                                    `gaagaaAGTCACCAAGTCCGTTAAGAAAGTCGTTAAGGAAGGGGCTCGTCCGGTTAAACAGGTTGCTGTCGGTCTAGCTGGTCTGGCTTGTGGTACTGGTGAAGCACAGATGGTGGAAGTACCACAAGCTGCCGCACAGATTGTTGACGTACCTGAGAAAGAGGTTTCCACTGAGGACGAAGCACAGCCAGAAAGCGGACGCAAGAAAGCTCGTGCTGTCGGTAAGAAATCCTTGAGTGTAGCCCGTAGCTCCGGTGGCGGTATCAACATTTAATCAGGAGGTTATCGTTGAAGACTGCAt                                >  1:616473/1‑301 (MQ=255)`  
                                                                                                                                                                                                                                                                                         `aaGTCACCAAGTCCGTTAAGGAAGTCGTTAAGGAAGGGGCTCGTCCGGTTAAACAGGTTGCTGGCGGTCTAGCTGGTCTGGCTGGTGGTACTGGTGAAGCACAGATGGTGGAAGTACCACAAGCTGCCGCACAGATTGTTGACGTACCTGAGAAAGAGGTTTCCACTGAGGACGAAGCACAGACAGAAAGCGGACGCAAGAAAGCTCGTGCTGGCGGTAAGAAATCCTTGAGTGTAGCCCGTAGCTCCGGTGGCGGTATCAACATTTAATCAGGAGGTTATCGTGGAAGACTGCATTGAAt                           <  1:375261/301‑1 (MQ=255)`  
                                                                                                                                                                                                                                                                                         `aaGTCACCAAGTCCGTCAAGAACGCCGTGAAGGCAGGGGTTCGTCCGGTTAAACAGGTTGCTGGCGGTCTAGCTGGTCTGGCTGGTGGTACTGGTGAAGCACAGATGGTGGAAGTACCACAAGCTGCCGCATAGATTGTTGACGTACCTGAGCAAGAGGTTTCCACTGAGGACGAAGCACAGACAGAAAGCGGACGCAAGAAAGCTCGTGCTGGCGGTAAGAAATCCTTGAGTGTAGCCCGTAGCTCCGGTGGCGGTATCAACATTTAATCAGGAGGTTATCGTGGAAGACTGCATTGAAt                           <  1:528791/301‑1 (MQ=255)`  
                                                                                                                                                                                                                                                                                              `cccAAGTCCGTTAAGAAAGTCGTTAAGGAAGGGGCTCGTCCGGTTATACAGGTTGCTGGCGGTCTAGCTGGTCTGGCTGGTGGTACTGGTGAAACACAGATGGTGGAAGTACCACAAGCTGCCGCACAGATTGTTGACGTACCTGAGAAAGAGGTTTCCACTGAGGACGAAGCACAGACAGAAAGCGGACGCAAGAAAGCTCGTGCTGGCGGTAAGAAATCCTTGAGTGTAGCCCGTAGCTCCGGTGGCGGTATCAACATTTAATCAGGAGGTTATCGTGGAAGACTGCATTGAATGGAcc                      <  1:698570/300‑1 (MQ=255)`  
                                                                                                                                                                                                                                                                                                   `acaggTTAAGAAAGTCGTTAAGGAAGGGGCTCGTCCGGTTAAACAGGTTGCTGGCGGTCTAGCTGGTCTGGCTGGTGGTACTGGTGAAGCACAGATGGTGGAAGTACCACAAGCTGCCGCACAGATTGTTGACGTACCTGAGAAAGAGGTTTCCACTGAGGACGAAGCACAGACAGAAAGCGGACGCAAGAAAGCTCGTGCTGGCGGTAAGAAATCCTTGAGTGTAGCCCGTAGCTCCGGTGGCGGTATCAACATTTAATCAGGAGGTTATCGTGGAAGACTGCATTGAATGGACCGGAgg                 <  1:155957/297‑1 (MQ=255)`  
                                                                                                                                                                                                                                                                                                            `gAAAGTCGTTAAGGAAGGGGCTCGTCCGGTTAAACAGGTTGCTGGCGGTCTAGCTGGTCTGGCTGGTGGTACTGGTGAAGCACAGATGGTGGAAGTACCACAAGCTGCCGCACAGATTGTTGACGTACCTGAGAAAGAGGTTTCCACGGAGGACGAAGCACAGACAGAAAGCGGACGCAAGAAAGCTCGTGCTGGCGGTAAGAAATCCTTGAGTGTAGCCCGTAGCTCCGGTGGCGGTATCAACATTTAATCAGGAGGTTATCGTGGAAGACTGCATTGAATGGACCGGAGGTGTCAActc        <  1:281142/301‑1 (MQ=255)`  

                                                                                                                                                                                                                                                                                                            `|`                                                                                                                                                                                                                                                                                                                    
`GTACATTCTGGACTCTACCGGAAAACATGTAGCGTACTTCGCGTGGTGCGTAAGCTGTGACATTCACCACAAAGGAGACATTCTGGATGTAACGTCCGTTGTCATTAATCCTGAGGCAGACTCTAAGGGCTTACAGCGATTCCTAGCGAAACGCTTTAAGTACCTTGCGGAACTCCACGATTGCGATTGGGTGTCTCGTTGTAAGCATGAAGGCGAGACAATGCGTGTATACTTTAAGGAGGTATAAGTTATGGGTAAGAAAGTTAAGAAGGCCGTGAAGAAAGTCACCAAGTCCGTTAAGAAAGTCGTTAAGGAAGGGGCTCGTCCGGTTAAACAGGTTGCTGGCGGTCTAGCTGGTCTGGCTGGTGGTACTGGTGAAGCACAGATGGTGGAAGTACCACAAGCTGCCGCACAGATTGTTGACGTACCTGAGAAAGAGGTTTCCACTGAGGACGAAGCACAGACAGAAAGCGGACGCAAGAAAGCTCGTGCTGGCGGTAAGAAATCCTTGAGTGTAGCCCGTAGCTCCGGTGGCGGTATCAACATTTAATCAGGAGGTTATCGTGGAAGACTGCATTGAATGGACCGGAGGTGTCAACTCTAAGGG  >  NC_001604.1/19285‑19891`

  

Alignment Legend

Aligned base mismatch/match (shaded by quality score): `ATCG/ATCG` `< 3 ≤` `ATCG/ATCG` `< 11 ≤` `ATCG/ATCG` `< 25 ≤` `ATCG/ATCG` `< 36 ≤` `ATCG/ATCG` `< 38 ≤` `ATCG/ATCG`

Unaligned base: `atcg`    Masked matching base: `atcg`    Alignment gap: `‑`    Deleted base: `‑`

Reads not counted as support for junction

read\_name Not counted due to insufficient overlap past the breakpoint.

read\_name Not counted due to not crossing MOB target site duplication.
